# Aberrant Metabolism in Hepatocellular Carcinoma Provides Diagnostic and Therapeutic Opportunities

**DOI:** 10.1155/2018/7512159

**Published:** 2018-11-04

**Authors:** Serena De Matteis, Andrea Ragusa, Giorgia Marisi, Stefania De Domenico, Andrea Casadei Gardini, Massimiliano Bonafè, Anna Maria Giudetti

**Affiliations:** ^1^Biosciences Laboratory, Istituto Scientifico Romagnolo per lo Studio e Cura dei Tumori (IRST) IRCCS, Meldola, Italy; ^2^Department of Engineering for Innovation, University of Salento, Lecce, Italy; ^3^CNR Nanotec, Institute of Nanotechnology, via Monteroni, 73100 Lecce, Italy; ^4^Italian National Research Council, Institute of Sciences of Food Production (ISPA), Lecce 73100, Italy; ^5^Department of Medical Oncology, Istituto Scientifico Romagnolo per lo Studio e Cura dei Tumori (IRST) IRCCS, Meldola, Italy; ^6^Department of Experimental, Diagnostic & Specialty Medicine, Alma Mater Studiorum, University of Bologna, Bologna, Italy; ^7^Department of Biological and Environmental Sciences and Technologies, University of Salento, Lecce, Italy

## Abstract

Hepatocellular carcinoma (HCC) accounts for over 80% of liver cancer cases and is highly malignant, recurrent, drug-resistant, and often diagnosed in the advanced stage. It is clear that early diagnosis and a better understanding of molecular mechanisms contributing to HCC progression is clinically urgent. Metabolic alterations clearly characterize HCC tumors. Numerous clinical parameters currently used to assess liver functions reflect changes in both enzyme activity and metabolites. Indeed, differences in glucose and acetate utilization are used as a valid clinical tool for stratifying patients with HCC. Moreover, increased serum lactate can distinguish HCC from normal subjects, and serum lactate dehydrogenase is used as a prognostic indicator for HCC patients under therapy. Currently, the emerging field of metabolomics that allows metabolite analysis in biological fluids is a powerful method for discovering new biomarkers. Several metabolic targets have been identified by metabolomics approaches, and these could be used as biomarkers in HCC. Moreover, the integration of different omics approaches could provide useful information on the metabolic pathways at the systems level. In this review, we provided an overview of the metabolic characteristics of HCC considering also the reciprocal influences between the metabolism of cancer cells and their microenvironment. Moreover, we also highlighted the interaction between hepatic metabolite production and their serum revelations through metabolomics researches.

## 1. Introduction

Hepatocellular carcinoma (HCC) is the most common type of primary liver cancer. It represents the fifth most common cancer worldwide and the second most frequent cause of cancer-related deaths [1]. HCC occurs most often in people with chronic liver diseases related to viral (chronic hepatitis B and C), toxic (alcohol and aflatoxin), metabolic (diabetes, hemochromatosis, and nonalcoholic fatty liver disease), and immune (autoimmune hepatitis and primary biliary) factors [1].

Effective management of HCC depends on early diagnosis and proper monitoring of the patients' response to therapy through the identification of pathways and mechanisms that are modulated during the process of tumorigenesis. In this context, the interest towards the concept of tumor metabolism is growing for several reasons: (i) metabolic alteration is a recognized hallmark of cancer, (ii) oncogenes drive alterations in cancer metabolism, (iii) metabolites can regulate gene and protein expressions, and (iv) metabolic proteins and/or metabolites represent diagnostic and prognostic biomarkers [2–6].

Metabolic alterations constitute a selective advantage for tumor growth, proliferation, and survival as they provide support to the crucial needs of cancer cells, such as increased energy production, macromolecular biosynthesis, and maintenance of redox balance. Although this is a common feature for all tumor types, it is still not completely clear how the tumor metabolic demand can really influence the metabolic profile and homeostasis of other tissues. Can they act as tumor bystanders or do they have an active role in supporting tumor growth? In this scenario, the liver represents a perfect metabolic model that governs body energy metabolism through the physiological regulation of different metabolites including sugars, lipids, and amino acids [7]. How can HCC metabolic alterations support tumor growth and influence systemic metabolism?

In this review, we take a detailed look at the alterations in intracellular and extracellular metabolites and metabolic pathways that are associated with HCC and describe the functional contribution on cancer progression and metabolic reprogramming of tumor microenvironment including immune cells. The analysis of circulating metabolites by metabolomics may provide us with novel data about this systemic crosstalk.

## 2. Reprogramming of Glucose Metabolism: Increased Uptake of Glucose and Lactate Production

In physiological conditions, the liver produces, stores, and releases glucose depending on the body's need for this substrate. After a meal, blood glucose enters the hepatocytes via the plasma membrane glucose transporter (GLUT). Human GLUT protein family includes fourteen members which exhibit different substrate specificities and tissue expression [8]. Once inside the cell, glucose is first converted, by glycolysis, into pyruvate and then completely oxidized into the mitochondrial matrix by the tricarboxylic acid (TCA) cycle and the oxidative phosphorylation. Alternatively, it can be channelled in the fatty acid synthesis pathway through the *de novo* lipogenesis (*DN*L). Glucose-6-phosphate dehydrogenase, the rate-limiting enzyme of the pentose phosphate pathway, is used in the liver to generate reduced nicotinamide adenine dinucleotide phosphate (NADPH) that is required for lipogenesis and biosynthesis of other bioactive molecules. In pathological conditions, glucose energy metabolism is altered. Important changes have been observed not only in the expression of specific transporters and enzyme isoforms but also in the flux of metabolites.

**Figure 1:**
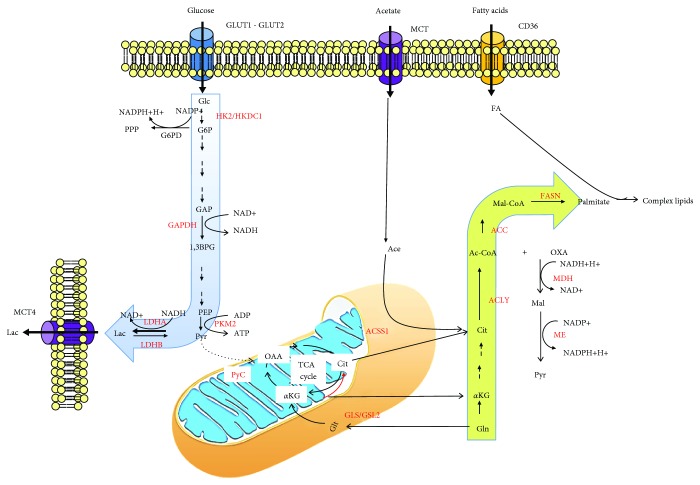
Metabolic reprogramming in HCC. Glucose enters the cancer cell via glucose transporters 1 and 2 (GLUT1 and GLUT2), and it is mainly used in the glycolytic pathway due to the overexpression of enzymes such as hexokinase 2 (HK2) and hexokinase domain containing 1 (HKDC1), glyceraldehyde-3-phosphate dehydrogenase (GAPDH), and pyruvate kinase 2 (PK2). The glycolytic pathway mainly produces, by the overexpression of lactate dehydrogenase A (LDHA) isoform, lactate (Lac) which is transported outside of the cell mainly throughout the monocarboxylate transporter isoform 4 (MCT4). Fatty acids enter cancer cells thanks to the upregulation of fatty acid translocase CD36. Nevertheless, fatty acid synthesis can also start from acetate (Ace), which is transported in the cell by MCT and converted into acetyl-CoA (Ac-CoA) by the mitochondrial isoform of acetyl-CoA synthase 1 (ACSS1). Moreover, glutamine (Gln) takes part in lipid synthesis after conversion into glutamate by mitochondrial glutaminase enzymes (GLS/GLS2). Glutamate is then converted into *α*-ketoglutarate (*α*KG) which can enter the tricarboxylic acid (TCA) cycle. Alternatively, *α*KG can undergo a reductive carboxylation by which it is transformed in citrate in the mitochondria (red arrow) or in the cytosol. In glutamine-free conditions, pyruvate (Pyr) can be converted into oxaloacetate (OAA) by the anaplerotic reaction catalysed by pyruvate carboxylase (PyC) enzyme. The *de novo* fatty acids synthesis is increased in cancer cells, and it is associated with a high expression of key enzymes such as acetyl-CoA carboxylase (ACC) and fatty acid synthase (FASN). This latter metabolic pathway is associated to a high production of reducing equivalents in the form of reduced nicotinamide adenine dinucleotide phosphate (NADPH) that is mainly produced in the first reaction of the pentose phosphate pathway (PPP) catalysed by glucose-6-phosphate dehydrogenase (G6PD) and by the malic enzyme (ME).

HCC tumors display a high level of glucose metabolism [9] (Figure 1). This enhanced metabolic demand is important for metabolic imaging and well supported by the ability of 18F-fludeoxyglucose (18F-FDG) positron emission tomography (PET) to correlate with unfavorable histopathologic features [10] and with the proliferative activity of tumor [11]. Moreover, high glucose levels as observed in patients with diabetes can accelerate tumorigenesis in HCC cells by generating advanced glycation end-products and O-GlcNAcylation of the Yes-associated protein (YAP) and c-Jun [12, 13].

The common feature of these alterations is an increased glucose uptake and production of lactate even in the presence of oxygen and fully functioning mitochondria (Warburg effect) [2, 3], but it is not correlated with enhanced gluconeogenesis as the expression of phosphoenolpyruvate carboxykinases 1 and 2 and fructose 1,6-bisphosphatase 1 (FBP1), has been reported to be downregulated in HCC [14]. Overall, this metabolic reprogramming promotes growth, survival, proliferation, and long-term maintenance [15]. To respond to this metabolic requirement, HCC tumors enhance glucose uptake [16] by upregulating GLUT1 and GLUT2 isoforms [17–19]. siRNA-mediated abrogation of GLUT1 expression inhibits proliferative and migratory potential of HCC cells [20], while GLUT2 overexpression was correlated to a worse prognosis [21, 22].

Once inside the cell, glucose is converted in glucose-6-phosphate (G6P) by the hexokinase (HK), which is the first enzyme of the glycolytic pathway. Five major hexokinase isoforms are expressed in mammalian tissues and denoted as HK1, HK2, HK3, HK4, and the isoform hexokinase domain containing 1 (HKDC1). HCC tumors express high levels of the HK2 isoform, and its expression is correlated with the pathological stage of the tumor [23, 24]. In HCC, HK2 knockdown inhibited the flux of glucose to pyruvate and lactate, increased oxidative phosphorylation, and sensitized to metformin [24]. Moreover, HK2 silencing also synergized with sorafenib to inhibit tumor growth in mice [24]. Interestingly, a new member of the HK family the isoform HKDC1 was upregulated in HCC tissues compared with the adjacent normal tissues. HCC patients with high expression levels of HKDC1 had poor overall survival. Silencing HKDC1 suppressed HCC cell proliferation and migration *in vitro*, probably by the repression of the Wnt/*β*-catenin pathway [25].

The transition to glucose metabolism involves alterations in different glycolytic enzymes. A well-characterized example is the decreased expression of FBP1 [26]. FBP1 downregulation by promoter methylation and copy-number loss contributed to HCC progression by altering glucose metabolism [26]. An increased expression of glyceraldehyde-3-phosphate dehydrogenase (GAPDH) [27, 28] and pyruvate kinases 2 (PKM2) [9, 29, 30] was also described. The altered expression of these enzymes supports the flux of glucose in the glycolytic pathway leading to the generation of pyruvate that may be either used to generate lactate or be directed towards the TCA cycle. The high glutamine utilization and the high levels of lactate observed in HCC tissues are both in accordance with the first hypothesis, suggesting an increased TCA carbon anaplerosis in HCC cells [24, 31]. Glutamine represents the most abundant amino acid in blood and tissues and represents the major hepatic gluconeogenic substrate. A metabolic shift towards glutamine regulates tumor growth in HCC [14]. Hepatoma cells have an accelerated metabolism and net glutamine consumption, with potential implication at the systemic level [32].

Glutamine is metabolized in several distinct pathways. By glutaminolysis, glutamine can be converted to *α*-ketoglutarate to replenish TCA cycle thus supporting energy production and providing intermediates for other biosynthetic pathways. By reductive carboxylation, glutamine moves in reverse of the TCA cycle from *α*-ketoglutarate to citrate to sustain lipid synthesis. So far, conflicting data have been reported on the role of glutamine in HCC. In fact, a study demonstrated that glutamine is metabolized mainly via glutaminolysis and not via reductive carboxylation to be converted into lactate [24]. Another study did not support this hypothesis as the great majority of enzymes involved in the conversion of glutamine to *α*-ketoglutarate were significantly downregulated in HCC compared to the normal liver [14]. This highlights the heterogeneous behaviour of this pathway that might be influenced by specific conditions of the microenvironment or its correlation to specific genetic alterations. For instance, glutamine metabolism in HCC varied in relation to the initiating lesion. Mouse liver tumors induced by MYC overexpression significantly increased both glucose and glutamine catabolism, whereas MET-induced liver tumors used glucose to produce glutamine [33].

The metabolic reaction that generates lactate from pyruvate is catalysed by lactate dehydrogenase (LDH). In humans, five active LDH isoenzymes are present and each of which is a tetrameric enzyme composed of two major subunits, M and H (formally A and B), encoded by *Ldh-A* and *Ldh-B*, respectively. The M subunit is predominantly found in the skeletal muscle, whereas the H subunit in the heart. LDHA and LDHB are upregulated in human cancers and associated with aggressive tumor outcomes [34–36].

In human HCC, LDHA expression was upregulated as a consequence of the downregulation of the microRNA-383 [37]. LDHA knockdown induced apoptosis and cell growth arrest in HCC cells and suppressed metastasis in a xenograft mouse model [38]. Serum LDH has been used as a prognostic indicator for patients with HCC treated with sorafenib, undergoing transcatheter arterial chemoembolization (TACE), or curative resection [39–42].

Lactate, the product of LDH activity, is exported in the extracellular milieu by monocarboxylate transporters (MCT). In HCC samples, an overexpression of MCT4 has been reported [43] and was also associated with Ki-67 expression [44]. Basigin, a transmembrane glycoprotein also called CD147, was found to be involved in the reprogramming of glucose metabolism in HCC cells. In particular, CD147 promoted glycolysis and facilitated the cell surface expression of MCT1 and lactate export [45]. Interestingly, blocking CD147 and/or MCT1 was reported to suppress HCC proliferation [45].

At a metabolic level, a high level of lactate with a low level of glucose was detected by nuclear magnetic resonance analysis in HCC samples [46]. This confirms the glycolytic shift toward lactate and provides a correlation between enzymatic alterations and metabolite expression [46]. The increased lactate concentration observed in the serum of HCC patients, compared to normal subjects, seems to be a consequence of this metabolic change [47]. However, the role of this secreted lactate still remains largely unclear. Nevertheless, increased lactate production was observed in patients with steatosis and steatohepatitis (NASH) compared to normal patients suggesting that the shift toward and anaerobic glucose metabolism can be involved in the first steps of liver carcinogenesis [48].

## 3. Altered Anabolic and Catabolic Lipid Pathways in HCC

The liver plays a key role in the metabolism of lipids and lipoproteins, and the anomalies in these metabolic pathways underlie HCC pathogenesis as demonstrated by increased risks observed in patients with obesity [49], diabetes [50], and hepatic steatosis [51]. After a carbohydrate reach meal, fatty acids can be synthetized from glycolytic pyruvate by *DN*L. Thus, entering the mitochondria, pyruvate is converted into acetyl-CoA by the pyruvate dehydrogenase enzyme. In the mitochondrial matrix, acetyl-CoA condensates with oxaloacetate to form citrate which, in conditions of high energetic charge, is conveyed to the cytoplasm throughout the citrate carrier for lipid synthesis. Key enzymes of cytosolic *DN*L are acetyl-CoA carboxylase (ACC), which catalyses the ATP-dependent carboxylation of acetyl-CoA to malonyl-CoA, and the multifunctional enzyme fatty acid synthase (FASN), which utilizes malonyl-CoA for synthesizing palmitoyl-CoA [52]. *DN*L also needs reducing power in the form of NADPH + H^+^, which is mainly generated through the glucose metabolism in the pentose phosphate pathway and in the malic enzyme reaction. *DN*L alterations were observed in HCC samples [53] and in other liver diseases, including nonalcoholic fatty liver disease (NAFLD) [54]. A combinatorial network-based analysis revealed that many enzymes involved in *DN*L, as well as enzymes related to NADPH production, such as glucose-6-phosphate dehydrogenase and malic enzyme, were upregulated in HCC with respect to the noncancerous liver samples [14].

During cancer progression, an overexpression of FASN is important for promoting tumor cell survival and proliferation [54], and it was also associated with poor patient prognosis [55]. In line with what observed in other types of tumors, recent studies described a functional association among lipogenesis, FASN, sterol regulatory element-binding protein-1 (SREBP-1), a transcription factor regulating FASN expression, and HCC [56–64]. The therapeutic effects of targeting FASN were investigated in several works. For instance, HCC induced by AKT/c-Met was fully inhibited in FASN knockout mice [65].

Alternative carbon sources can support the generation of acetyl-CoA required for *DN*L. This can derive from exogenous acetate, which is transported into cells by members of the MCT family and then converted to acetyl-CoA by acetyl-CoA synthase enzymes (mitochondrial ACSS1 and ACSS3 or cytosolic ACSS2) to fuel fatty acid synthesis (Figure 1). Mitochondrial ACSS1 expression, but not ACSS2 or ACSS3 ones, is significantly upregulated in HCC compared to noncancerous liver and associated with increased tumor growth and malignancy under hypoxic conditions [14].

Tumors can be addicted or independent from *DN*L by the activation of complementary pathways. In fact, both *de novo* synthetized and exogenous fatty acids can support the growth of HCC tumors [66]. The studies performed on animal models demonstrated that the inhibition of lipogenesis via genetic deletion of ACC genes increased susceptibility to tumorigenesis in mice treated with the hepatocellular carcinogen diethylnitrosamine, demonstrating that lipogenesis is essential for liver tumorigenesis [67].

The liver is able to take up nonesterified fatty acids from the blood, in proportion to their concentration, either via specific transporters (fatty acid transport protein (FATP) or fatty acid translocase/CD36) or by diffusion. The activation of the CD36 pathway has been associated with tumor aggressiveness by the induction of the epithelial-mesenchymal transition (EMT) program [68], which is a process that contributes to cancer progression [69, 70]. This is mediated through the involvement of specific pathways. The analysis of the Cancer Genome Atlas (TGCA) dataset revealed a significant association between CD36 and EMT markers, potentially by the activation of Wnt and TGF-*β* signaling pathways [71].

The liver is able to oxidize fatty acids by both mitochondrial and peroxisomal *β*-oxidation. The entry of fatty acids into the mitochondria is regulated by the activity of the enzyme carnitine palmitoyltransferase-I (CPT-I) [72], which catalyses the synthesis of acylcarnitines from very long acyl-CoA and carnitine, thus allowing the entry of polar fatty acids in the mitochondrial matrix. In a rat model of NASH, a decreased mitochondrial CPT-I activity [73] and dysfunction of both complex I and II of the mitochondrial respiratory chain [74] have been demonstrated. Moreover, deregulation of mitochondrial *β*-oxidation with downregulation of many enzymes involved in fatty acid oxidation has been reported in HCC patients [14]. Accordingly, the urinary level of short- and medium-chain acylcarnitines was found to be different in HCC vs. cirrhosis, and butyrylcarnitine (carnitine C4:0) was defined as a potential marker for distinguishing between HCC and cirrhosis [75]. All together, these data indicate that mitochondrial alterations can represent an early determinant in HCC.

The liver represents also a major site of synthesis and metabolism of endogenous cholesterol. The pool of cholesterol is tightly regulated, and it reflects the input of cholesterol from the diet, its biosynthesis, the secretion and uptake of cholesterol from plasma lipoproteins, the conversion of cholesterol into bile, and the reuptake of biliary cholesterol and bile acids from the intestine to the liver. The rate-limiting enzyme in the cholesterol synthesis is the 3-hydroxy-3-methylglutaryl-CoA reductase, which catalyses the synthesis of mevalonate.

There is increasing evidence that the mevalonate pathway is implicated in the pathogenesis of HCC [76, 77]. To this respect, clinical studies demonstrated that statins, widely used to reduce cholesterol levels, were able to reduce the risk of HCC [78] and showed antiproliferative effects *in vitro* on HepG2 cells and *in vivo* on rats with HCC [79]. Data from a meta-analysis report that the use of statins could significantly cut the risk of liver cancer and that fluvastatin is the most effective drug for reducing HCC risk compared to other statin interventions [80].

Cholesterol is also used in the liver for the synthesis of bile acids, which are hydroxylated steroids that, once secreted in the intestine, provide for solubilisation of dietary cholesterol, lipids, fat-soluble vitamins, and other essential nutrients, thus promoting their delivery to the liver. Primary bile acids, such as cholic acid and chenodeoxycholic acid, are synthesized in hepatocytes and can be conjugated to glycine or taurine [81]. Cholic acid conjugated to glycine, in the form of glycocholic acid, represents a secondary bile acid that is synthesized by microbiota in the small intestine [81]. It has been reported that intrahepatic bile acid may have a stimulatory effect of hepatic tumorigenesis [82] and abnormal bile acid metabolism has been correlated with HCC [83–86]. The hepatic deregulation of bile acid metabolism can result in increased serum level of glycolic acid, as reported by Guo and collaborators [87].

Modification in the activity of enzymes related to phospholipid remodelling has been reported in a rat model of cirrhosis [88]. Moreover, an altered lipid metabolism, including phospholipids, fatty acids, and bile metabolites, was observed in serum samples from HCC patients [89, 90]. In particular, higher phosphatidylcholines (PC) concentrations were observed in HCC patients at early and late stage compared to cirrhotic and control subjects, indicating a disturbance of the phospholipid catabolism [85]. This result significantly correlated with higher levels of PC observed at tissue level [91].

## 4. Alterations in Amino Acid and Protein Metabolism Underlying HCC

The liver carries out many functions in protein metabolism. A broad spectrum of proteins responsible for the maintenance of hemostasis, oncotic pressure, hormone, lipid transport, and acute phase reactions are synthetized in the liver. Among these proteins, albumin is synthesized almost exclusively by the liver, and alone, it accounts for 40% of hepatic protein synthesis. Moreover, the liver is also able to synthetize thyroid-binding globulin, VLDL apoB 100, and complement.

A very recent work reports that patients with lower serum albumin levels have significantly larger maximum tumor diameters, greater prevalence of portal vein thrombosis, increased tumor multifocality, and higher *α*-fetoprotein levels with respect to patients with higher albumin levels. These data indicate that decreased serum albumin correlates with increased parameters of HCC aggressiveness, therefore, having a role in HCC aggressiveness [92].

In the liver, the synthetized nonessential amino acids (AA) play a pivotal role in the maintenance of diverse homeostatic functions such as gluconeogenesis. Both synthetized amino acids and those derived from the diet are utilized either for protein synthesis or catabolized (except branched chain amino acids) by transamination or oxidative deamination reactions. These processes produce keto acids that can be oxidized to produce energy in the form of ATP. Several enzymes used in these pathways (for example, alanine transaminase and aspartate transaminase) are commonly assayed in serum to assess liver damage. Moreover, the oxidative deamination of amino acids produces ammonium ions, a toxic product whose detoxification can either occur in extrahepatic tissues, throughout the synthesis of glutamine when combined with glutamate, or in the liver, to make urea which is then transported to the kidneys where it can be directly excreted in urine.

An increased AA metabolism is generally observed in human tumors, in line with the role of AA and enzymes responsible for their production in cancer initiation and progression [93–96]. The shift toward an increased amino acid production is considered as a consequence of the described altered glucose metabolism. In the condition of increased consumption of glucose by aerobic glycolysis, amino acids can be used as glucose precursors or activators of glycolytic enzymes [97].

An altered AA metabolism characterizes HCC compared to other liver diseases. For instance, serum levels of alanine, serine, glycine, cysteine, aspartic acid, lysine, methionine, tyrosine, phenylalanine, tryptophan, and glutamic acid were dramatically increased in HCC compared with healthy subjects, together with a lower ratio of branched-chain amino acids (valine, leucine, and isoleucine) to aromatic amino acids (tyrosine, phenylalanine, and tryptophan) [98, 99].

Furthermore, AA bioavailability not only contributes to anabolic and catabolic pathways but it is also essential during HCC pathogenesis by supporting cellular hypoxic responses [100].

## 5. Oxidative Metabolism Imbalance in HCC

Oxidative stress occurs when reactive oxygen species (ROS) production overwhelms the normal antioxidant capacity of cells [101]. ROS are short-lived and very reactive molecules that rapidly react with cellular biomolecules yielding oxidatively modified products that eventually lead to cell injury and death. Due to the high instability of ROS, they cannot be easily detected, and protein carbonyls, 8-hydroxydeoxyguanosine, and 4-hydroxynonenal (which are oxidatively modified products of proteins, DNA, and lipids, respectively) have been widely used as markers for oxidative stress [102].

Accumulating evidence suggests that many types of cancer cells have increased levels of oxidative stress and ROS production with respect to normal cells [103]. As a consequence of this, redox homeostasis is finely regulated in cancer cells with an underappreciated role in the control of cell signaling and metabolism. For instance, ROS-mediated inhibition of PKM2 allows cancer cells to sustain antioxidant responses by diverting glucose flux into the pentose phosphate pathway and increasing the production of reducing equivalents for ROS detoxification [104]. Understanding the mechanisms at the basis of ROS homeostasis might have therapeutic implications.

Oxidative damage is considered a key pathway in HCC progression and increases patient vulnerability for HCC recurrence [105]. Oxidative stress closely correlates with HCV- and NASH-related HCC, but relatively weakly with HBV-related HCC [106]. Moreover, it has been reported that NASH-related HCC patients had a diminished serum antioxidative function compared with nonalcoholic fatty liver disease patients [107].

Glutathione is a nonenzymatic tripeptide that plays a central role in the cellular antioxidant defense system, and it represents the most abundant antioxidant in hepatocytes. Glutathione is synthesized intracellularly from cysteine, glycine, and glutamate, and it is abundantly found in the cytosol and mitochondria and in a smaller percentage in the endoplasmic reticulum [108]. During glutathione antioxidant function, the reduced form of glutathione (GSH) is oxidized to the glutathione disulfide dimer (GSSG). The regeneration of the reduced form requires the enzymatic action of glutathione reductase and reducing equivalents in the form of NADPH + H^+^. A significant increase in all amino acids related to the GSH synthesis, including 5-oxoproline, was observed in the serum of HCC patients, and an increase of G6P that represents an important source of NADPH for the generation of GSH has been also reported [109]. Moreover, signs of DNA and lipid oxidative damages were found in HCC. Indeed, an increased level of 8-hydroxydeoxyguanosine was found in chronic hepatitis, corresponding to an increased risk of HCC [110, 111]. In addition, a mass spectrometry study highlighted an increased level of 4-hydroxynonenal in human tissues of HCC compared to peripheral noncancerous tissues [112].

The GSH:GSSG ratio is considered an important indicator of the redox balance in cells, with a higher ratio representing low oxidative stress [107]. Upon depletion of GSH, ROS induce oxidative stress which causes liver damage, and reduced GSH levels have been reported in various liver diseases [113]. By-products of GSH synthesis are represented by *γ*-glutamyl peptides that are biosynthesized through a reaction of ligation of glutamate with various amino acids and amines by the action of *γ*-glutamylcysteine synthetase. Serum level of *γ*-glutamyl peptides, measured by capillary chromatography-MS/MS, was increased in patients with virus-related HCC [114, 115], and it was considered a reliable potential biomarker for this pathology [115]. The 2-hydroxybutyric acid is another compound considered in relation to GSH metabolism. It is primarily produced in mammalian hepatic tissues, which catabolize threonine or synthesize glutathione. Under conditions of intense oxidative stress, hepatic glutathione synthesis is increased, and there is a high demand for cysteine. In such cases, homocysteine is diverted from the transmethylation pathway and instead it is used to produce cystathionine, which is then cleaved into cysteine and finally incorporated into glutathione. 2-Hydroxybutyric acid is then produced from reduction of *α*-ketobutyrate, which is released as a by-product of the cystathionine conversion to cysteine. An increased serum concentration of 2-hydroxybutyric acid as well as of xanthine, an intermediate in the purine degradation process producing H_2_O_2_, and several *γ*-glutamyl peptides, were found in HCC with respect to control subjects [116, 117].

## 6. Metabolic Reprogramming of HCC Microenvironment

**Figure 2:**
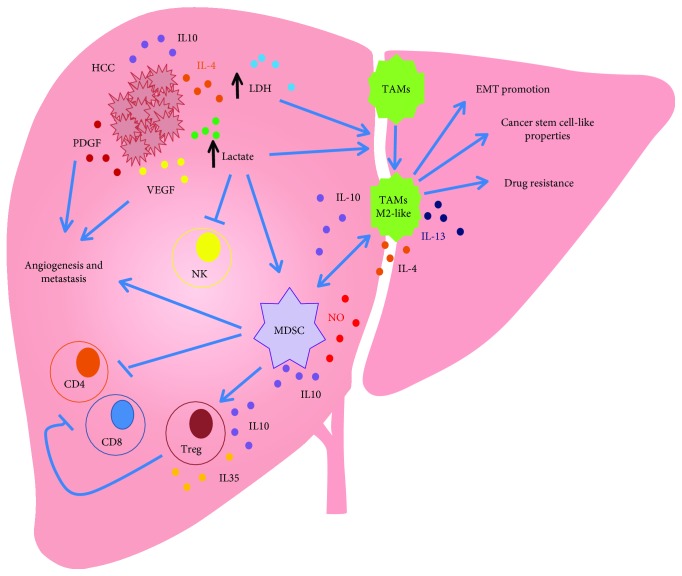
Metabolic connections between HCC and immune cells. Tumor-associated macrophages (TAM) and myeloid-derived suppressor cells (MDSC) are the major components of tumor-infiltrate and are abundant in HCC microenvironment, with a key role in supporting tumor initiation, progression, angiogenesis, metastasis, and drug resistance. HCC cells acquire an altered metabolism resulting in increased levels of LDH and lactate that, on the one hand, promote TAM polarization in M2-like phenotype, favoring the EMT, cancer stem cell-like properties, and drug resistance, and, on the other hand, increase the number of MDSC and inhibit NK cell function.

HCC microenvironment consists of stromal cells, hepatic stellate cells, and endothelial and immune cells. The crosstalk between tumor cells and their surrounding microenvironment is required for sustaining HCC development by promoting angiogenesis, EMT, or by modulating the polarization of immune cells. Tumor-associated macrophages (TAM) and myeloid-derived suppressor cells (MDSC) are the major components of tumor-infiltrate and are abundant in HCC microenvironment (Figure 2) [118–120]. Metabolites released from tumor cells can have an impact on immune cells.

Inflammatory stimuli promote the switching of macrophages towards an M1-like phenotype characterized by the production of inflammatory cytokines. On the contrary, anti-inflammatory stimuli induce these cells to acquire an M2-like phenotype with immunosuppressive functions [121]. Thus, during chronic inflammation, macrophages predispose a given tissue to tumor initiation by releasing themselves factors that promote neoplastic transformation. In the successive phases of inflammation, the macrophage phenotype shifts more toward one that is immunosuppressive and supports tumor growth, angiogenesis, and metastasis [122]. In this respect, both epidemiological and clinical studies have demonstrated that various chronic inflammatory diseases can predispose to increased risk of cancer at the same site of inflammation, and HCC is a clear example of inflammation-related cancer.

TAM originate from circulating monocytic precursors, and they are recruited to tumor cells by tumor-derived signals. It has been reported that, in the early phase of the tumor, TAM acquire an inflammatory phenotype and shift their metabolism toward an anaerobic glycolytic pathway [122, 123]. This allows polarized macrophages to rapidly fuel themselves with energy and to cope with hypoxic tissue microenvironment. The interplay between M2-like TAM and cancer is complex, and it is involved in each step of HCC development. It has been demonstrated that this TAM subset promotes migration and EMT. It also induces cancer stem cell-like properties and drug resistance in human HCC, thus highlighting the importance of targeting the immune microenvironment as a mechanism to inhibit HCC recurrence and metastasis (Figure 2) [124]. Moreover, it has been shown that an increase in the M2-macrophage population is associated with poor prognosis in HCC [125]. TAM can use metabolites released from cancer cells to modulate their polarization status. This is supported by the findings that demonstrated that tumor-released lactate is utilized by TAM to increase the expression of vascular endothelial growth factor and to induce an M2-like status [126]. Lactate can also have effects on other cell types by increasing the number of MDSC, thus inhibiting NK cell function (Figure 2) [127]. The immunosuppressive functions of MDSC can also be regulated by other metabolites, including fatty acids. In MDSC, increased fatty acid uptake and fatty acid oxidation are induced by a STAT3/STAT5-mediated pathway leading to an increased expression of CD36. Accumulation of lipids increases the oxidative metabolism of MDSC and activates their immunosuppressive mechanisms [128].

## 7. Concluding Remarks and Perspectives

Global changes in metabolic pathways were identified across different tumor types [129, 130]. The picture that emerged for the comparison between tumors and normal tissue revealed common demands in biochemical pathways associated with biomass production, such as glycolysis, pentose phosphate pathway, purine, and pyrimidine biosynthesis, irrespective of the cell of origin. This cancer tissue-specific metabolic signature is well defined in HCC, where the high glucose demand supports PPP pathway, lactate production by anaerobic glycolysis, and fatty acid production by FASN. Glucose utilization is regulated at multiple levels, including the transcriptional regulation of metabolic enzymes and transporters whose expression is altered in HCC tissues, and consistent with the identification of mitochondrial DNA alterations which was associated with a reduced oxidative phosphorylation and enhanced glycolysis [131].

HCC cells also rely on other carbon substrates to support anaplerotic pathways. A role for glutamine and acetate in sustaining HCC bioenergetics has been proposed. Alternative anaplerotic pathways exist that rely on the transformation of pyruvate to oxaloacetate by pyruvate carboxylase to support the growth of HCC cells in glutamine-free conditions [132]. The dependence of HCC cells from lipid uptake should be also considered, suggesting the existence of a metabolic flexibility and of a possible cross-talk between metabolic pathways that might result by changes imposed by the tumor microenvironment or by the activation of specific signaling pathways. HCC cells can, for instance, modulate fatty acids uptake by the upregulation of CD36 and caveolin 1 that is mediated by the activation of Wnt and TGF-*β* pathways.

More unique metabolic features were also defined by TGCA studies where significant alterations by mutation or downregulation by hypermethylation in albumin, apolipoprotein B, and carbamoyl phosphate synthase I metabolic genes were observed in HCC [71].

Overall, this metabolic program seems to be essential for HCC biology to sustain tumor growth and progression, and targeting of these pathways might have significant clinical implications. For instance, clinical effects of dexamethasone are explicated *in vivo* by restoring gluconeogenesis [133], inhibition of proline production and targeting of transketolase significantly enhances the cytotoxic effect of sorafenib *in vivo* [134], and insights into nucleotide and lipid metabolism of HCC may provide novel clinical opportunities [135].

However, the design of therapeutic metabolic targeted strategies should be carefully evaluated taking into account tumor heterogeneity and tumor interaction with the microenvironment. Several approaches were applied to address these complexities, including computational, proteomics, and metabolomics. Specifically, these methods tried to move forward the analysis of a single metabolite to obtain not a static snapshot of tumor biology but a more dynamic picture of metabolic changes in cancer. In this context, the field has expanded to bypass some classical limitations as the small number of samples to be analyzed and the number of analytes to be revealed. By the integration of different omics approaches, genome-scale metabolic models (GEMM) provided a way to study metabolic pathways at systems level [136] and to predict the action of a pathway inhibition at multiple layers of biological complexity. A personalized GEMM has been realized for NAFLD patients [137, 138] and HCC patients [14, 139] to characterize a specific disease-related metabolic phenotype. Moreover, the detailed analysis of a specific metabolic status provides the opportunity to study network perturbations after drug treatment. GEMM built from six HCC patients has been used to predict the action of antimetabolites on cancer growth. The model identified 147 antimetabolites that can inhibit growth in any of the studied HCC tumors, and a smaller group of antimetabolites that were predicted to be effective in only some of the HCC patients, showing a more personalized mechanism of action [139]. Moreover, GEMM has become an informative approach to elucidate tumor heterogeneity at the metabolic level [14].

Although most studies were centered on tumor tissue, metabolomics analyses at the blood level showed to be promising in predicting metabolic alterations at the tissue level. Solid results were obtained with some metabolites, such as lactate and AA, and their altered expression in serum clearly mirrors a metabolic alteration of tumor tissue. The same is probably true for other metabolites such as fatty acids but not yet clarified.
